# A Glycolipid‐Like Biosurfactant Molecule Derived From an *Exophiala spinifera* Strain With Significant Antibiofilm, Antifungal, and Antiquorum Sensing Activities

**DOI:** 10.1155/ijm/5630530

**Published:** 2026-07-23

**Authors:** Amit Yadav, Swathy Sadanandan Anand, Bipin Gopalakrishnan Nair, Jayashree Gopalakrishna Pai, Jayalekshmi Haripriyan, Sudarslal Sadasivan Nair

**Affiliations:** ^1^ Amrita School of Biotechnology, Amrita Vishwa Vidyapeetham, Kollam, Kerala, India, amrita.edu

## Abstract

A biosurfactant‐producing fungal strain, *Exophiala spinifera* CSF123, was isolated from cashew oil‐contaminated soil. Internal transcribed spacer sequencing followed by phylogenetic analysis revealed that the isolated fungus shared 98.42% similarity with *E. spinifera* CBS66.76. The biosurfactant molecule, named ES‐414, produced by the fungus was purified to homogeneity by HPLC, and subsequent electrospray mass spectrometric analysis showed that its neutral mass is 414 Da. The molecule displayed remarkable antibiofilm activity. At concentrations of 15 and 80 *μ*g/mL, ES‐414 showed 62.17% biofilm inhibition against *Candida tropicalis* and 66% inhibition against *Candida albicans*, respectively. It also displayed significant antifungal properties against *C. albicans*, *C. tropicalis*, *Candida glabrata*, *Candida parapsilosis*, and *Candida krusei*. ES‐414, in a dose‐dependent manner, inhibited the quorum sensing activity of *Chromobacterium violaceum*. Thermogravimetric analysis of ES‐414 indicated that the molecule remains stable up to 240°C. Detailed structural characterization using GC‐MS, FTIR, UV‐visible spectroscopy, elemental analysis, Molisch test, TLC analysis, and solubility studies indicated that ES‐414 is a glycolipid‐like molecule.

## 1. Introduction

The growing demand for sustainable technologies has intensified the search for natural alternatives to harmful synthetic chemicals. Synthetic surfactants, for example, raise concern due to their toxicity, persistence, and nonbiodegradability, posing significant environmental and health risks [1, 2]. Consequently, major attention has been shifted toward microbial‐derived biosurfactants due to their environmental compatibility, cost‐effectiveness, and stability to function under extreme conditions of temperature, pH, and salinity. Microbial biosurfactants are amphipathic molecules, which are released extracellularly [3, 4]. They decrease surface tension and improve emulsification by forming micelles at the interfaces of immiscible liquids, such as water and oil [5, 6]. Apart from surface‐active properties, biosurfactants exhibit antifungal, antibiofilm, antibacterial, and antiquorum sensing properties [7], offering a wide range of biotechnological applications in environmental and pharmaceutical sectors.

Even though biosurfactants from bacteria and fungi are extensively studied, there has been relatively less focus on characterizing biosurfactants from extremophilic organisms [8, 9]. The identified biosurfactants from fungi mainly come under four major classes, namely glycolipids, lipopeptides, fatty acids, and polymeric biosurfactants [10]. These molecules possess amphiphilic structures, comprising a hydrophilic group such as carbohydrates, amino acids, or phosphate and a hydrophobic moiety, usually long‐chain or hydroxylated fatty acids [11].

Glycolipids are composed of sugars and lipids with a carbohydrate moiety attached to a hydroxyl or aliphatic fatty acid [12]. Among glycolipids, sophorolipids, cellobiose lipids, and mannosylerythritol lipids are the most well‐known and have been demonstrated to exhibit antiviral, antibiofilm, and antibacterial properties against pathogens such as *Staphylococcus aureus* and *Candida albicans* [13–15].

Lipopeptides contain a hydrophobic tail, usually a fatty acid, and a hydrophilic head made up of amino acids. Fatty acids can have different degrees of branching [16]. Enniatin and echinocandin are among the well‐characterized lipopeptides. Fungal species such as *Aspergillus fumigatus* and *Fusarium* have been investigated for their potential to produce these compounds [17, 18].

When cultivated on alkanes, several microbes can biodegrade hydrocarbons and generate extracellular fatty acid biosurfactants. These saturated fatty acids, which span from C12 to C14, have hydroxyl groups and alkyl chains. *Penicillium spiculisporum* and *Talaromyces trachyspermus* are key producers of fatty acid biosurfactants [19].

Polymeric biosurfactants, on the other hand, are often heteropolymers of sugars, proteins, and lipids. The most studied polymeric biosurfactants include liposan and yasan. *Candida utilis* and *Yarrowia lipolytica* are well‐explored fungi for polymeric biosurfactants [20].

Biosurfactants derived from various fungal sources have shown diverse biotechnological applications, particularly in therapeutic and environmental fields. For instance, researchers extracted biosurfactants from *Aspergillus carneus* and *Aspergillus niger*, which exhibited significant antifungal and antibacterial properties against pathogens including *C. albicans*, *Candida tropicalis*, *S. aureus*, and *Salmonella typhi*, highlighting their immense potential for therapeutical applications [21–23]. Besides, biosurfactants are widely examined for their biofilm and quorum sensing inhibitory properties.

Biofilms are structured communities of microbial cells embedded within a self‐produced extracellular polymeric substance (EPS) matrix that adheres to biotic or abiotic surfaces. Biofilm formation is an important virulence mechanism that enhances microbial survival and resistance to antimicrobial agents. Quorum sensing regulates several biofilm‐associated processes, including adhesion, maturation, and virulence factor production. Disruption of biofilms and quorum sensing pathways has emerged as an efficient strategy against microbial pathogens [24, 25]. Several biosurfactants have exhibited disruption of biofilms and quorum sensing pathways [26–29]. Owing to their amphiphilic nature, biosurfactants alter surface properties, reduce microbial adhesion, and inhibit biofilm development. In addition to preventing initial microbial attachment, biosurfactants can promote biofilm dispersion by disrupting the structural integrity of the EPS matrix and altering cell‐surface hydrophobicity [30–32]. Biosurfactants have also been shown to interfere with quorum sensing regulated phenotypes such as pigment production, motility, and expression of virulence‐associated genes [33, 34]. In this context, the present study focuses on the isolation and structural and functional characterization of a biosurfactant from an *Exophiala* fungal isolate obtained from a cashew nut oil‐contaminated soil. The isolated molecule exhibits distinct structural features and shows remarkable antibiofilm, antifungal, and antiquorum sensing activity. The findings of this study enhance the understanding of novel fungal biosurfactant molecules and their potential for applications in therapeutical, industrial, and environmental challenges.

## 2. Materials and Methods

### 2.1. Isolation of Fungus

A soil sample contaminated by cashew nutshell oil was collected from a cashew factory premise at Karunagappally, Kollam, Kerala (India). For isolating the fungus, the soil sample was serially diluted and pour‐plated on Rose Bengal agar containing 0.1 mg/mL chloramphenicol (HiMedia, India) and incubated at 27°C for 7 days. The colonies obtained were maintained in a sterile potato dextrose plate as suggested by Olanbiwoninu et al. [35].

### 2.2. Determination of Biosurfactant Activity

#### 2.2.1. Production of Biosurfactants

The fungus was inoculated into 100 mL of potato dextrose broth (PDB) in Erlenmeyer flasks and incubated at 27°C with shaking at 120 rpm. The spent media was collected after 7 days of incubation and evaluated for the presence of biosurfactants.

#### 2.2.2. Oil Displacement Assay

The presence of biosurfactant molecules in the spent culture media was analyzed by an oil displacement assay as described by Ewida and Mohamed [36] with modifications. Briefly, 1 mL of used engine oil was added to the surface of 25 mL of distilled water in a petri dish to form a thin oil layer. A total of 20 *μ*L of the fungal spent media was then gently placed on the center of the oil layer. In the presence of biosurfactants, the oil will be displaced, resulting in the formation of a zone of clearance that indicates a positive result. Two percentage of sodium dodecyl sulfate (SDS) was used as a positive control.

#### 2.2.3. Emulsification Assay

The emulsifying capacity of fungal spent media was evaluated by emulsification index (E_24_) using kerosene and cashew nutshell oil. An equal volume of kerosene or cashew nutshell oil was added to fungal spent media in a test tube, vortexed at high speed for 2 min, and allowed to stand for 24 h. The percentage of the emulsification index was calculated using the equation: E_24_ = (height of emulsion formed × 100)/total height of solution, where E_24_ is the emulsification index after 24 h [37].

#### 2.2.4. Drop Collapse Assay

A drop collapse assay was done to further evaluate the biosurfactant activity. In brief, 20 *μ*L of used engine oil was added to the wells of a 96‐well microtiter plate and was equilibrated for 24 h at room temperature. A total of 20 *μ*L of fungal spent media was then added to the wells and observed for spreading of the drop over oil‐coated wells. Two percentage of SDS was taken as a positive control [38].

#### 2.2.5. Lipolytic Activity

Lipase production was detected on a potato dextrose agar (PDA) plate supplemented with 1% (v/v) tributyrin. The isolated fungus was streaked onto the plate and incubated at 27°C for 48–72 h. After the incubation, the plate was evaluated for the presence of a clearance zone around the colony, indicating the lysis of tributyrin [39].

### 2.3. Morphological and Molecular Identification of the Isolated Fungus With Biosurfactant Property

The isolated fungus was cultured on a PDA plate and incubated at 27°C for morphological characterization. After 7 days of growth, Gram staining was carried out [40] and observed at 100× magnification using a monocular microscope (LM‐52‐1602).

Genomic DNA from the fungus was isolated by using the NucleoSpin Plant II Kit (Macherey‐Nagel). ITS1F (TCCGTAGGTGAACCTGCGG) and ITS4R (TCCTCCGCTTATTGATATGC) primers were used for PCR amplification in an Applied Biosystems GeneAmp 9700 PCR System, followed by Sanger sequencing. The obtained sequence was compared with the related sequences through BLAST analysis in GenBank (NCBI), and a phylogenetic tree was constructed. Subsequently, the sequence was submitted to GenBank.

### 2.4. Extraction and Isolation of Biosurfactant Molecules From the Fungal Isolate

For the extraction of molecules with biosurfactant properties, the fungal culture was prepared in PDB and incubated at 27°C for 12 days. The mycelial biomass was separated from the blackish‐green culture medium by centrifugation at 17,696 g for 20 min using an Avanti J‐20 XP centrifuge (Beckman Coulter, United States). The resultant spent culture medium was acidified to a pH of 3 using 0.6 M HCl and was kept at room temperature for 24 h. The medium was then autoclaved at 120°C for 15 min to fully precipitate the dark pigment. The precipitated pigment was collected by centrifugation at 17,696 g for 10 min and washed with distilled water until neutral pH was achieved [41]. The final precipitate was lyophilized for downstream analysis.

### 2.5. High‐Performance Liquid Chromatography (HPLC)

Purification of the lyophilized sample was carried out by using HPLC. A total of 4 mg/mL of the sample dissolved in 1 N NaOH was filtered using a 0.2‐*μ*m filter. The purification process was carried out using an Agilent HPLC system (1260 Infinity II) equipped with an Agilent Zorbax SB‐C18 column (5 *μ*m, 9.4 × 150 mm). The mobile Phase A consisted of 100% water with 0.1% trifluoroacetic acid (TFA), and B contained 100% methanol with 0.1% TFA. The column was equilibrated with 50% methanol, and the molecules were eluted with a linear gradient from 50% to 100% methanol in 28 min. The flow rate was maintained at 1 mL/min, and the absorbance was monitored at 279 nm. The separated fractions that showed surfactant property were individually lyophilized.

### 2.6. Determination of Solubility of the HPLC‐Purified Biosurfactant

The solubility of the HPLC‐purified and lyophilized biosurfactant was assessed in different solvents, like deionized water, organic solvents such as acetone, chloroform, methanol, isopropanol, toluene, and dimethyl sulfoxide (DMSO), and an alkaline solution (1 N NaOH). In each experiment, 1 mg of the purified biosurfactant was mixed with 1 mL of the solvents. The solubility was assessed by visually observing the presence of particles [42].

### 2.7. Determination of Antiquorum Sensing Activity

The antiquorum sensing activity of the biosurfactant was evaluated in *Chromobacterium violaceum* ATCC 12472 by extracting and quantifying the violacein pigment as described by Sadik et al. [43]. Briefly, *C. violaceum* was cultured in LB broth with biosurfactant concentrations ranging from 250 to 125 *μ*g/*μ*L at 37°C for 24 h. A total of 1 mL of the bacterial culture from each concentration was centrifuged at 13,523 g for 10 min. The supernatant was aspirated, and 1 mL of DMSO was added to the pellet. The mixture was vortexed vigorously for 30 s to solubilize the violacein, then centrifuged again at 13,523 g for 10 min. The supernatant containing violacein was transferred to a microtiter plate, and absorbance was measured at 585 nm. The percentage of inhibition of violacein production in the presence of the biosurfactant was determined as % violacein inhibition = (OD_585_ of control – OD_585_ of treated/OD_585_ of control) × 100.

### 2.8. Antimicrobial Activity of the Biosurfactant

Antimicrobial activity of the biosurfactant was determined against three strains of bacteria (*Pseudomonas aeruginosa* PAO1, *S. aureus* MTCC 96, and *C. violaceum*) and five strains of fungi (*C. albicans* MTCC 189, *Candida parapsilosis* MTCC 1965, *C. tropicalis* MTCC 230, *Candida krusei* MTCC 9215, and *Candida glabrata* MTCC 3019) using an agar‐well diffusion assay. Briefly, LB agar and PDA plates were prepared for bacteria and fungi, respectively, and wells were made in the agar using a sterile borer. The purified biosurfactant weighing 500 *μ*g was added to each well, and the plates were then incubated overnight at 37°C. Following incubation, the antimicrobial activity was assessed by measuring the zones of inhibition around the wells. For comparison, 100 *μ*g fluconazole and ciprofloxacin (HiMedia, India) were used as positive controls for bacteria and fungi, respectively. The zones of inhibition produced by the pigment were compared with the positive control to determine the relative antimicrobial potency [44].

### 2.9. Determination of Minimum Inhibitory Concentration (MIC)

The MIC of the biosurfactant against different fungal strains was assessed using the broth dilution method. In 96‐well microplates, 100 *μ*L of PDB containing fungal cultures adjusted to an optical density (OD) of 0.2 at 600 nm was added to each well. The biosurfactant was added at concentrations ranging from 30 to 250 *μ*g/mL and serially twofold diluted across the wells for each fungal isolate. The plates were incubated at 37°C for 24 h. After incubation, the MIC was determined by spotting 5 *μ*L of culture from each well onto PDA plates to check for fungal growth [45].

### 2.10. Antibiofilm Activity

The antibiofilm activity of the biosurfactant was evaluated using a crystal violet–based microplate assay against *C. albicans*, *C. parapsilosis*, *C. tropicalis*, *C. krusei*, and *C. glabrata*. Briefly, fungal cultures grown in PDB were adjusted to an OD of 0.2 at 600 nm and were added to a 96‐well microplate, followed by the addition of the biosurfactant at concentrations based on the respective fungal isolate′s MIC value. The plates were incubated for 24 h at 37°C. After incubation, planktonic cells were carefully removed, and the wells were washed twice with PBS (pH 7.2). Biofilms were stained with 120 *μ*L of 0.2% crystal violet solution for 20 min and washed thoroughly with PBS to remove the unbound dye. The bound stain was then solubilized using 100 *μ*L of 33% acetic acid, and the absorbance was measured at 595 nm to quantify the biofilm. Further to visualize biofilm inhibition, biofilms were developed on sterile glass coverslips at MIC/2 concentration of the biosurfactant for the respective fungal isolate and subsequently examined using ZEISS Primo Star microscope at 100× magnification [46].

### 2.11. Surface Tension Analysis

For determining the surface tension, a clean glass vessel containing 30 mL of the biosurfactant was mounted on the force tensiometer (KSV Sigma 701) platform. A clean platinum Wilhelmy plate was used as the probe, and the surface tension analysis was performed.

### 2.12. UV‐Visible Spectroscopy

The HPLC‐purified biosurfactant was dissolved in 100% DMSO, and its ultraviolet‐visible spectrum in the range of 200–700 nm was measured using a Shimadzu UV‐1800 spectrophotometer.

### 2.13. Fourier Transform Infrared (FTIR) Spectroscopy

The FTIR spectrum ranging from 4000 to 450 cm^−1^ was obtained using a Perkin Elmer FTIR spectrometer (Spectrum Two). The lyophilized biosurfactant was thoroughly mixed with IR‐grade potassium bromide, and the resulting spectra were recorded.

### 2.14. Elemental Composition Analysis

Elemental composition analysis of the purified biosurfactant was carried out using a Thermo Fisher Scientific FlashSmart elemental analyzer.

### 2.15. Thermogravimetric Studies

Thermogravimetric analysis (TGA), differential thermal analysis (DTA), and derivative thermogravimetry (DTG) were conducted using a Hitachi STA7000 system. Approximately 10 mg of the purified biosurfactant was used to determine its thermal stability. The biosurfactant was subjected to a controlled temperature range, starting from 40°C and increasing up to 740°C, which provided comprehensive information about the decomposition behavior and thermal characteristics.

### 2.16. Qualitative Analysis of Carbohydrate and Lipid Moieties of the Biosurfactant

The presence of carbohydrate moiety was determined using the Molisch test. Briefly, 2 mg/mL of the purified biosurfactant was mixed with Molisch reagent in a test tube, and concentrated sulfuric acid was carefully added along the side of the test tube. The reaction mixture was then observed for development of a violet ring at the interface of acid and biosurfactant. Sucrose was used as a positive control. The presence of lipid moiety in the biosurfactant was determined by thin‐layer chromatography (TLC). Briefly, the purified biosurfactant was subjected to TLC using a solvent system consisting of toluene:ethyl acetate:formic acid (5:4:1, v/v/v). Following chromatographic separation, the TLC plate was exposed to iodine vapors for lipid visualization. Cholesterol was used as a positive control for comparison.

### 2.17. LC‐MS Analysis of the Biosurfactant

LC‐MS analysis of the HPLC‐purified biosurfactant was conducted using an Agilent 6540 series electrospray ionization quadrupole time‐of‐flight (ESI‐QTOF) mass spectrometer coupled to an Agilent 1290 uHPLC system. The purified biosurfactant was characterized in both positive and negative ionization modes to determine its neutral mass.

### 2.18. GC‐MS Analysis

The biosurfactant was analyzed using GC‐MS (Agilent 5977B system) to identify its constituent compounds and determine their molecular weights. The oven temperature was initially set at 80°C and held for 2 min, then ramped at 10°C per minute to a final temperature of 300°C.

### 2.19. Statistical Analysis

All experiments were performed in triplicate, and the results are presented as the mean ± standard deviation (SD). Statistical significance was evaluated using one‐way ANOVA. Data visualization and graphing were performed using GraphPad Prism software (Version 10.5.0).

## 3. Results

### 3.1. Isolation of Fungus From Cashew Nutshell Oil‐Contaminated Soil and Screening for Biosurfactant Activity

Four distinct fungal colonies were grown in a PDA plate inoculated with the serially diluted soil sample. Each colony was then picked and maintained as a pure culture and labeled as CSF123, CSF223, CSF323, and CSF423. The isolates were screened for biosurfactant production using various assays.

Initially, an oil displacement assay was conducted as described in Section 2.2.2 [47]. Among the four fungal isolates, only CSF123 displayed a positive outcome for the oil displacement assay with a prominent zone of clearance (Figure S1A), indicating the presence of biosurfactant in the spent media of CSF123, suggesting the ability of this isolate to produce surface‐active compounds.

### 3.2. Confirmation of Biosurfactant Activity

#### 3.2.1. Determination of Emulsification Index of the Fungal Spent Media

The emulsification assay further confirmed the observed biosurfactant activity of the fungal isolate CSF123, using cashew nutshell oil and kerosene oil as substrates. The spent media from the *Exophiala spinifera* CSF123 culture showed an emulsification index (E_24_) of 33.3% with cashew nut oil and 66.67% with kerosene oil. These obtained values were comparable with the positive control (2% SDS), which served as a standard for emulsification efficiency, with an E_24_ of 58.6% for kerosene and 53.33% for cashew nut oil (Figure S1B).

#### 3.2.2. Drop Collapse Assay

A drop collapse assay was also performed to confirm the biosurfactant activity of CSF123, where the fungal spent media was placed on an oil‐coated surface to observe whether it would spread over the surface or remain as an intact drop. The positive result, indicated by the collapse and spreading of the drop, demonstrated the presence of surface‐active molecules capable of reducing the surface tension (Figure S1C). This further confirms the fungal isolate CSF123′s ability to produce biosurfactants.

### 3.3. Qualitative Determination of Lipolytic Activity of the Fungal Isolate *E. spinifera* CSF123

The lipolytic activity of the fungal isolate *E. spinifera* CSF123 was examined by growing it on PDA plates supplemented with 1% tributyrin as the substrate. A clear zone of lipolysis around the colony was observed, indicating the positive lipolytic activity (Figure S1D). This observation suggests that the fungus not only produces biosurfactants but also possesses the ability to release enzymes that break down lipid substrates, further supporting its industrial applications, such as biodegradation and bioremediation [48].

### 3.4. Morphological and Molecular Identification of the Fungal Isolate

The fungal isolate *E. spinifera* CSF123 exhibited distinct morphological characteristics when grown on PDA plates. The colonies were mucoid in texture with a circular margin and had a raised structure. The unique dark greenish‐black coloration of the colonies (Figure S2A) is indicative of the fungal isolate′s pigment production. Microscopic analysis of the isolate following Gram staining revealed that the fungus predominantly displayed a yeast‐like form with the presence of pseudohyphae (Figure S2B).

DNA from the fungal isolate was then successfully isolated using the NucleoSpin Plant II Kit. The PCR product obtained after amplification with ITS primers was purified and sequenced. The sequence generated had 442 nucleotides, and its positions were analyzed and aligned using BioEdit software, Version 7.2. Comparison of the sequence with the databank using BLAST analysis showed 98.42% similarity with the *E. spinifera* strain CBS66.76. The data were subsequently deposited in the NCBI GenBank as *E. spinifera* CSF123 with an accession number of OR462372. A phylogenetic tree was then constructed to analyze the evolutionary relationship of our isolate with the other closely related *Exophiala* species in the database (Figure S2C). The analysis revealed variation in the sequence of fungus we isolated compared with the known sequences.

### 3.5. Isolation of Biosurfactant Molecule From *E. spinifera* CSF123

The isolated fungus exhibited dark greenish‐black pigmented colonies on PDA agar plates. To extract the pigment, the fungus was cultured in PDB, and the pigment was recovered by acid precipitation followed by autoclaving to enhance pigment precipitation. The precipitate was washed with distilled water until the pH reached neutral, and it was then lyophilized to produce a dry pigment. From 500 mL of the culture, a total of 40 mg of pigment was recovered. This efficient yield indicates that the fungus produces significant amounts of the pigment, potentially as a secondary metabolite, that may have biological or industrial applications.

### 3.6. HPLC Purification

**Figure 1:**
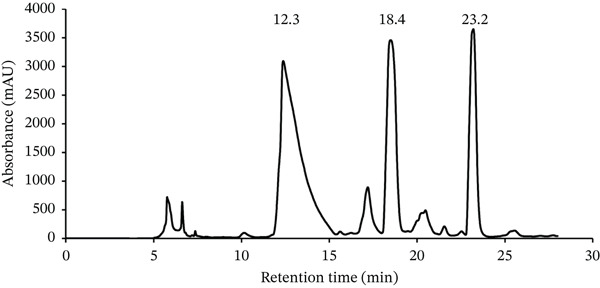
HPLC purification of the *Exophiala spinifera* CSF123 spent medium components after acid precipitation, autoclaving, and lyophilization. Out of the three major peaks, the peak at 18.4 min was selected for subsequent characterization.

The pigment retrieved through the previous step (as outlined in Section 3.5) was further subjected to HPLC purification by using a reversed‐phase column. The pigment, dissolved in 1 N NaOH, was effectively separated into three major fractions with retention times of 12.3, 18.4, and 23.2 min, as illustrated in Figure 1. The fraction eluted at 18.4 min was of particular interest due to its prominent biosurfactant property. This fraction was lyophilized and subjected to various structural and functional characterizations.

### 3.7. LC‐MS Analysis

**Figure 2:**
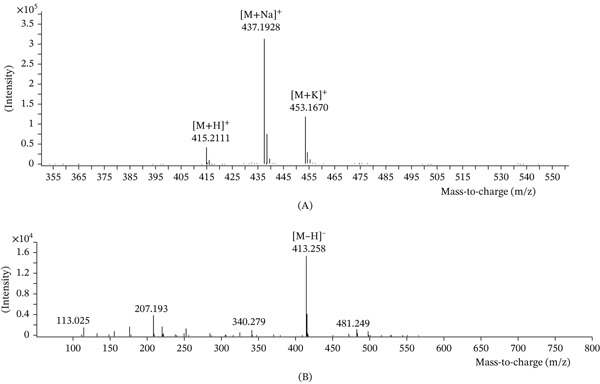
LC‐ESI MS profiles of the biosurfactant molecule, named as ES‐414, isolated from *Exophiala spinifera* CSF123 spent medium. (A) Mass spectrum of the HPLC‐purified ES‐414 recorded in positive ionization mode, showing a peak at 415.2111 m/z, indicating [M + H]^+^ ion. The peaks at 437.1928 and 453.1670 m/z, respectively, are the [M + Na]^+^ and [M + K]^+^ ions. (B) The mass spectrum of the purified ES‐414 recorded in negative ionization mode displays a peak at 413.258 m/z, demonstrating the [M−H]^−^ ion.

To determine the molecular mass, the purified biosurfactant, corresponding to the HPLC retention time of 18.4 min, was subjected to LC‐ESI MS characterization. In the positive ionization mode, we observed three peaks at 415.2111, 437.1928, and 453.1670 m/z (Figure 2A). The peaks, respectively, are presumed to be formed from protonation ([M + H]^+^), sodiation ([M + Na]^+^), and potassiation ([M + K]^+^). The sample, however, in the negative ionization mode generated a prominent peak at 413.258 m/z (Figure 2B), possibly due to a deprotonation ([M−H]^−^). Based on these observations, the neutral mass of the biosurfactant was determined to be around 414 Da. With the molecular information, we have named the isolated biosurfactant from *E. spinifera* CSF123 as ES‐414.

### 3.8. Solubility Analysis of the HPLC‐Purified ES‐414

The solubility profile of the purified ES‐414 was determined using different solvents, including water and organic solvents. We found that ES‐414 was insoluble in common solvents like pure water, acetone, chloroform, toluene, and methanol but soluble in isopropanol, DMSO, and alkaline water. The observed solubility profile is notably distinct from the typical biosurfactants isolated from different biological sources, including fungi [49, 50].

### 3.9. Biosurfactant Activity of ES‐414

**Figure 3:**
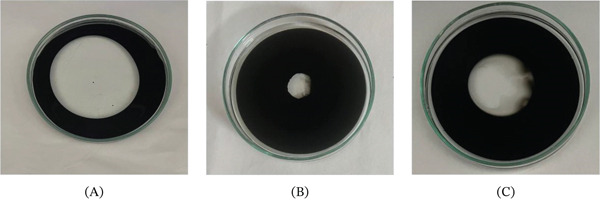
Oil displacement assay indicating the biosurfactant activity of the HPLC‐purified ES‐414 extracted from *Exophiala spinifera* CSF123. (A) Oil displacement zone produced by 20 *μ*g of ES‐414 dissolved in 20 *μ*L DMSO, exhibiting a clear displacement zone of 40 mm. (B) Negative control consisting of 20 *μ*L DMSO. (C) Positive control consisting of 20 *μ*g sodium dodecyl sulfate (SDS) dissolved in 20 *μ*L DMSO, exhibiting a displacement zone of 30 mm.

To evaluate its biosurfactant activity, the purified ES‐414 was subjected to an oil displacement assay (Figure 3). The addition of the solution containing 20 *μ*g of ES‐414 to the oil–water interface resulted in a significant zone of clearance of 40 mm, whereas the positive control, SDS with 20 *μ*g of concentration, displayed a value of 30 mm. The result strongly suggests that ES‐414 is contributing to the biosurfactant activity that the fungus exhibits. We further confirmed the surfactant potential of ES‐414 through surface tension analysis. A total of 1 mg/mL of HPLC‐purified sample significantly reduced the surface tension of alkaline water (1 N NaOH) from 70 to 50.80 ± 0.19 mN/m. The reduction in surface tension indicates that the ES‐414 possesses surface‐active properties, further supporting its role as a biosurfactant.

### 3.10. Antimicrobial Activity of ES‐414

**Figure 4:**
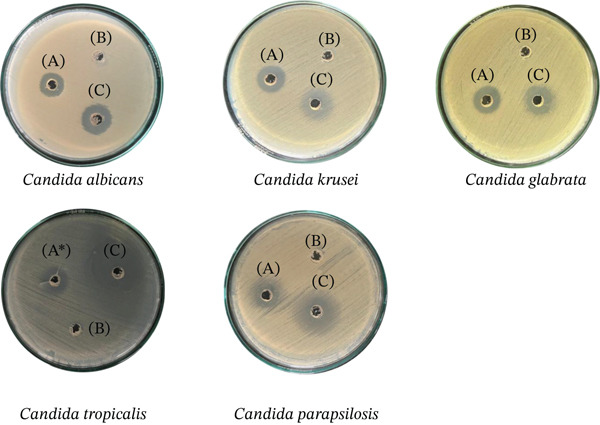
Agar well diffusion assay illustrating the antifungal activity of ES‐414 against *Candida albicans*, *Candida krusei*, *Candida glabrata*, *Candida tropicalis*, and *Candida parapsilosis*. Wells contained (A) fluconazole (100 *μ*g), (A∗) fluconazole (500 *μ*g), (B) DMSO, and (C) purified ES‐414 (500 *μ*g).

ES‐414 was analyzed for its antimicrobial properties against bacterial and fungal strains. Although it did not demonstrate antibacterial activity against *P. aeruginosa*, *S. aureus*, and *C. violaceum* (Figure S3). ES‐414 exhibited significant antifungal properties against *C. albicans*, *C. tropicalis*, *C. glabrata*, *C. parapsilosis*, and *C. krusei* by inhibiting the growth of all fungal strains, thereby indicating the antifungal activity of the biosurfactant (Figure 4). ES‐414 produced a 15 mm inhibition zone against *C. albicans*, with an MIC value of 80 *μ*g/mL. Among the non‐*albicans* strains, the lowest MIC was recorded against *C. tropicalis* (15 *μ*g/mL), which also showed a 15‐mm zone of inhibition. This was followed by *C. glabrata*, *C. krusei*, and *C. parapsilosis*, each showing a 16‐mm inhibition zone with MIC values of 60, 100, and 125 *μ*g/mL, respectively (Table 1). The lowest MIC observed against *C. tropicalis* suggests comparatively stronger antifungal activity against this strain.

**Table 1:** Antifungal activity of ES‐414 against *Candida albicans* and non‐*albicans* strains. The amount of ES‐414 used for zone of inhibition studies was 500 *μ*g, and that of the control (fluconazole) was 100 *μ*g. For *Candida tropicalis*, 500 *μ*g fluconazole was used.

Fungal strain	Zone of inhibition (in mm) shown by ES‐414	Zone of inhibition (in mm) shown by fluconazole	MIC (*μ*g/mL)
*Candida albicans*	19	15	80
*Candida parapsilosis*	18	16	125
*Candida tropicalis*	31	15	15
*Candida krusei*	16	16	100
*Candida glabrata*	17	16	60

### 3.11. Antiquorum Sensing Activity

**Figure 5:**
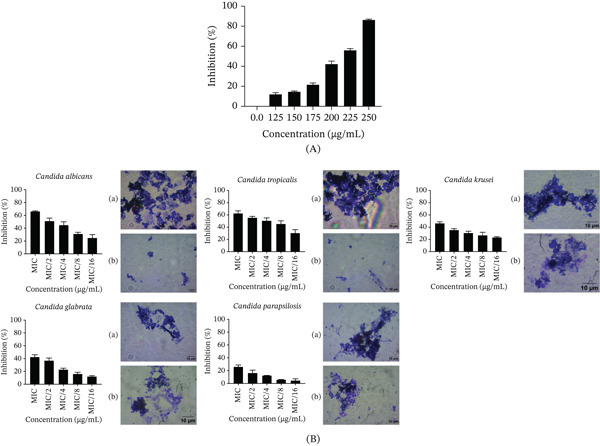
(A) Percentage inhibition of violacein pigment production in *Chromobacterium violaceum* in a dose‐dependent manner, demonstrating the antiquorum sensing activity of the biosurfactant ES‐414. Results are shown as the mean ± standard deviation of three independent experiments. Statistical analysis was performed using Dunnett′s test, showing a significant difference between treated samples and the control (∗∗∗∗*p* < 0.001 vs. control). (B) Percentage inhibition of biofilm formation by ES‐414 against *Candida albicans* and non‐*albicans* strains at MIC and sub‐MIC concentrations. Each value corresponds to the mean ± standard deviation obtained from three independent experiments. Microscopic visualization of biofilm formation at 100× magnification, showing (a) untreated biofilm and (b) biofilm inhibition by ES‐414 at MIC/2 concentration.

ES‐414 was assessed for its antiquorum sensing activity against *C. violaceum.* A promising inhibition in violacein pigment production was found in a dose‐dependent manner upon treatment with the biosurfactant, without any bactericidal or bacteriostatic effect, which is an indicator of the antiquorum sensing activity. At concentrations of 150, 200, and 250 *μ*g/mL, the biosurfactant inhibited violacein production by 14%, 41%, and 86%, respectively, indicating the antivirulent potential of the biosurfactant produced by *E. spinifera* CSF123 (Figure 5A).

### 3.12. Antibiofilm Activity of ES‐414

The ability of the biosurfactant, ES‐414, to inhibit biofilm formation by opportunistic fungal pathogens *C. albicans*, *C. parapsilosis*, *C. krusei*, *C. glabrata*, and *C. tropicalis* was evaluated using the crystal violet assay. These species are major contributors to biofilm‐mediated systemic infections [51]. The biosurfactant at a concentration of 80 *μ*g/mL demonstrated 66% inhibition against *C. albicans*, which is well‐known for robust biofilm formation and resistance to antimicrobial treatments. Among the non‐*albicans* strains, *C. tropicalis* showed the highest inhibition of 62% at 15 *μ*g/mL of the biosurfactant. This species is known for its high resistance to antifungal drugs, largely due to its robust extracellular matrix [52]. The lowest inhibition of 25% was recorded against *C. parapsilosis* at 125 *μ*g/mL, whereas approximately 40% inhibition was observed against *C. krusei* and *C. glabrata* at concentrations of 100 and 60 *μ*g/mL, respectively (Figure 5B).

### 3.13. UV‐Visible Spectroscopic Analysis of ES‐414

**Figure 6:**
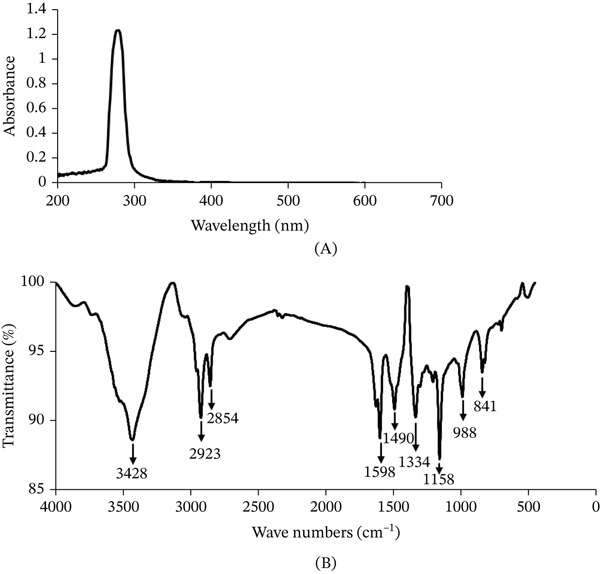
(A) UV‐visible spectrum of the HPLC‐purified ES‐414 from *Exophiala spinifera* CSF123. The spectrum was recorded from 200 to 700 nm, showing a maximum absorption peak at 279 nm. (B) FTIR vibrational spectrum of ES‐414.

UV‐visible spectroscopic analysis of ES‐414 was conducted to gain perceptions into its chemical characteristics. We recorded the spectrum in the 200–700 nm range, revealing a maximum absorbance at 279 nm, which then declined from the UV to the visible region (Figure 6A). The sharp peak at 279 nm shows that ES‐414 has aromatic conjugated structures that can absorb UV radiation and protect cells from oxidative damage and UV exposure [53].

### 3.14. FTIR Characterization

FTIR spectroscopy was employed to further characterize ES‐414. The FTIR spectra, as shown in Figure 6B, revealed several key features that provide insights into the chemical composition and structural characteristics of the biosurfactant. The intense absorption band in the 3428 cm^−1^ region indicates the stretching vibrations of polymeric OH groups and the NH stretching vibrations [54]. The observation indicates the presence of hydroxyl and amine functional groups, which are characteristic of biosurfactants and play a key role in mediating molecular interactions. The absorption bands at 2923 and 2854 cm^−1^ are attributed to the CH_3_ and CH_2_ aliphatic groups, indicating the presence of alkyl chains in the biosurfactant structure [55] contributing to the biosurfactant′s overall molecular framework. The strong absorption peak at 1598 cm^−1^ corresponds to the bending vibration modes of aromatic ring C=C or C=N bonds. This peak is indicative of the presence of conjugated aromatic structures [56]. The peaks at 1490 and 1334 cm^−1^ can be, respectively, attributed to C‐C stretching and the presence of phenol groups [57, 58] The peaks below 1000 cm^−1^ are associated with the out‐of‐plane bending vibrations of aromatic alkene (C‐H) bonds [59]. This feature highlights the presence of aromatic rings, which are integral to the biosurfactant′s structure and functionality.

### 3.15. Elemental Composition Analysis

Elemental composition analysis of the purified biosurfactant offered helpful information about its chemical makeup. The analysis revealed the following elemental percentages: carbon (C) at 28.14%, hydrogen (H) at 2.77%, nitrogen (N) at 2.02%, oxygen (O) at 46.43%, and sulfur (S) at 0.35%. The carbon content of the isolated biosurfactant is relatively low when compared with typical biosurfactants, where it is usually higher and lies in the 40%–60% range. The lower carbon percentage of the *E. spinifera* CSF123‐derived biosurfactant could be due to its distinct molecular structure.

### 3.16. Thermogravimetric Studies

**Figure 7:**
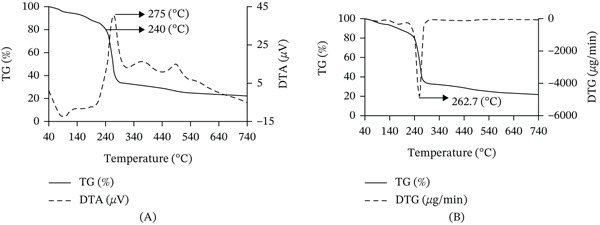
(A) Thermogravimetric analysis (TGA) of ES‐414, demonstrating thermal stability up to 240°C, with 80% of the mass retained, and differential thermal analysis (DTA) showing an exothermic peak at 275°C, indicating the onset of decomposition. (B) Derivative thermogravimetry (DTG) depicting a pronounced peak at 262.7°C, corresponding to the major decomposition phase of ES‐414.

TGA of ES‐414 was performed to assess its thermal stability and decomposition characteristics. The TGA data indicate that the biosurfactant remains stable up to 240°C, with 80.9% of the original mass retained. This high degree of mass retention at elevated temperatures suggests that ES‐414 possesses substantial thermal stability and is resistant to decomposition at higher temperatures. The DTA revealed a significant exothermic peak at 275°C. This peak suggests the onset of rapid decomposition of the polymer, marking a critical temperature at which the biosurfactant undergoes substantial thermal degradation (Figure 7A). The DTG data exhibited a pronounced peak at 262.7°C (Figure 7B), with a peak rate of mass loss of 4859 *μ*g/min. This peak corresponds to the major decomposition phase observed in the TGA, highlighting the temperature at which ES‐414 undergoes its most rapid breakdown. The peak rate of mass loss indicates the point of maximum thermal instability, providing a precise temperature for the most significant phase of decomposition. The TGA results also indicated that approximately 21% of the original mass remained at 740°C. This residual mass reflects the presence of thermally stable carbonaceous material, suggesting that ES‐414 contains carbonaceous components that remain intact even at high temperatures [60].

### 3.17. Qualitative Determination of Carbohydrate and Lipid Moieties in ES‐414

The purified biosurfactant ES‐414 was tested for the presence of carbohydrates using the Molisch test, which exhibited the formation of a violet‐colored ring at the interface between the acid and the biosurfactant solution (Figure S4A) indicating the presence of a carbohydrate moiety in the biosurfactant.

The presence of lipids in the biosurfactant was confirmed by TLC. Upon exposure to iodine vapors, yellowish‐brown bands were observed (Figure S4B), indicating the presence of a lipid component in ES‐414. The observations together denote that ES‐414 is a glycolipid‐like biosurfactant [61].

### 3.18. GC‐MS Analysis

The purified biosurfactant was subjected to GC‐MS analysis to possibly identify the biosurfactant molecule. Results illustrated a prominent peak at 124 m/z (Figure S5).

## 4. Discussion

Fungal biosurfactants are natural compounds with diverse biological activities and functional properties. Researchers have isolated multiple biosurfactant‐producing fungal strains from diverse environments, each exhibiting unique attributes with potential industrial applications. This study specifically explores the biosurfactant potential of *E. spinifera* CSF123, a strain isolated from a cashew oil‐contaminated soil. To the best of our knowledge, the present study is the first one to reveal biosurfactant production by *E. spinifera* and subsequent structural characterization and assessment of the biosurfactant′s potential applications. Generally, fungi are known to synthesize four major classes of biosurfactants: glycolipids, lipopeptides, fatty acids, and polymeric biosurfactants. Glycolipids, lipopeptides, and fatty acid–based biosurfactants are comprised of low–molecular‐weight molecules, with masses typically ranging from 500 to 1500 Da [62]. The masses of glycolipids in this group are usually between 550 and 700 Da, and those of lipopeptides fall between 800 and 1500 Da [63]. Some fungi, such as *C. lepus*, reported to secrete free fatty acid molecules as biosurfactants, have masses lower than 500 Da [64]. In contrast, polymeric biosurfactants are high–molecular‐weight biosurfactants, with masses ranging from 5000 to over 750,000 Da [65]. In this study, we isolated a biosurfactant named ES‐414 from *E. spinifera* CSF123. MS characterization of the biosurfactant revealed a molecular mass of 414 Da, which differs from that of known glycolipid and lipopeptide classes of biosurfactant molecules. Further, GC‐MS analysis of ES‐414 revealed a prominent peak at 124 m/z (Figure S4). The observed peak, we presume, corresponds to a specific fragment ion from a fatty acid component as reported by Zargar et al. [66].

When it comes to solubility, glycolipids and fatty acids are soluble in polar organic solvents like methanol, ethanol, and chloroform and show increased solubility in alkaline solutions like KOH or NaOH [67, 68]. Quite interestingly, ES‐414, though soluble in alkaline solutions, remains insoluble in solvents such as methanol, ethanol, acetone, chloroform, and toluene. It is, however, observed to be soluble in DMSO and isopropanol. Notably, the presence of carbohydrate and lipid moieties, indicated by the Molisch test and TLC analysis, together with solubility analysis suggests ES‐414 is likely to possess a glycolipid‐like structure.

Our UV‐visible spectroscopic studies indicated that ES‐414 had the highest absorbance at 279 nm, which is in line with what Nicula et al. [69] communicated about the spectral properties of lipoproteins. However, lipoproteins bear masses much higher than ES‐414, and neither the LC‐MS nor the GC‐MS profiles of ES‐414 indicate the presence of amino acids, as seen with lipoproteins. These findings, together with the solubility behavior, indicate that the biosurfactant molecule produced by *E. spinifera* CSF123 has a distinct structure but also shares features with glycolipids. Nevertheless, FTIR spectroscopic characterization of ES‐414 identified key features such as broad absorption bands for OH and N‐H stretching and peaks for aromatic rings and C=O stretching, consistent with chemical structures of glycolipids [70].

Thermogravimetric characterization of ES‐414 indicated that the molecule remains stable up to 240°C, with a significant decomposition peak at 262.7°C and a residual mass of approximately 20% at 740°C. These correlations emphasize the potential of biosurfactant usage in harsh industrial, medical, and environmental conditions without the loss of its activity. Notably, the observed thermal stability and decomposition behavior of ES‐414 are similar to glycolipid‐like biosurfactants as reported by Ribeiro et al. [71]. However, the elemental composition analysis of ES‐414 revealed a distinct chemical profile, with a remarkably low carbon content. This is a vital observation, as it diverges from the typical carbon‐rich nature of hitherto known biosurfactants [72]. A detailed NMR study might shed light on the unique structure of ES‐414 and clarify the ambiguities.

ES‐414 demonstrated significant antiquorum sensing activity against a widely used quorum sensing biomonitor strain, *C. violaceum*, as evidenced by its inhibition of violacein pigment production. Violacein synthesis in *C. violaceum* is regulated by an acyl‐homoserine lactone (AHL)–mediated quorum‐sensing system; therefore, the reduction in pigment production indicates that ES‐414 interferes with the quorum‐sensing signaling mechanism. [73]. At a concentration of 250 *μ*g/mL, ES‐414 achieved more than 80% inhibition, highlighting its ability to disrupt cell‐to‐cell communication possibly by inhibiting the signaling molecules involved in quorum sensing pathways. Notably, ES‐414 demonstrated stronger activity compared with other reported biosurfactants. For example, while the biosurfactant molecule isolated by Adnan et al. [74] showed 60% inhibition of the violacein pigment at 1.25 mg/mL of its concentration, the molecule identified by Patel et al. [75] yielded 60% inhibition at a concentration of 1.5 mg/mL. Similarly, the biosurfactant isolated by Raissa et al. [76] displayed 61% inhibition at 256 *μ*g/mL of its concentration.

ES‐414 also displayed strong antifungal and antibiofilm properties against *C. albicans* and multiple non‐*albicans* strains. *C. albicans* is known for forming biofilms on various biotic and abiotic surfaces. Upon treatment with ES‐414 at MIC (80 *μ*g/mL), over 60% inhibition of *C. albicans* biofilm was observed, which is much more promising than other reported studies. Abruzzo et al. [77], for instance, reported only 50% inhibition of *C. albicans* biofilms at 1.25 mg/mL of a biosurfactant they isolated. Another study by Watchaputi et al. [78] demonstrated 50% inhibition of mature *C. albicans* biofilms at 256 *μ*g/mL of the biosurfactant. Among the non‐*albicans* strains tested, ES‐414 demonstrated approximately 60% inhibition of biofilm (at an MIC value of 15 *μ*g/mL) against *C. tropicalis*, an emerging pathogen primarily responsible for superficial and invasive infections in immunocompromised patients in hospital settings [79]. ES‐414′s antibiofilm potential against *C. tropicalis* surpasses the findings of numerous reported studies. As demonstrated in an article by Madduri et al. [80], one of the molecules they isolated possesses nearly 50% biofilm inhibition at its 16 *μ*g/mL concentration, and the sophorolipid isolated by Haque et al. [81] required a substantially higher concentration of 120 *μ*g/mL to show 80% biofilm inhibitory property against *C. tropicalis*. The pronounced inhibition of biofilms suggests that ES‐414 may interfere with key stages of biofilm development, including initial adhesion, microcolony formation, and maturation. Given its antiquorum sensing activity, the observed biofilm inhibition of ES‐414 could potentially be associated with its ability to disrupt quorum‐sensing–mediated communication, which is known to play a crucial role in different stages of biofilm formation [82].

Nevertheless, despite exhibiting strong antifungal activity, ES‐414 lacks antibacterial properties, suggesting its specific mechanism of action against fungal pathogens. Overall, our results confirm that the *Exophiala*‐derived biosurfactant shares multiple identical characteristics with the glycolipid class of biosurfactants. The presence of carbohydrate and lipid, as indicated by the Molisch test and TLC analysis, suggests the glycolipid‐like nature of ES‐414. Interestingly, even though ES‐414 exhibited increased solubility in alkaline solutions, DMSO, and isopropanol, which is similar to the solubility profile of typical glycolipids, it remains insoluble in most organic solvents, including chloroform, methanol, acetone, ethanol, and toluene, diverging from glycolipid behavior [83, 84].

However, the UV absorbance, molecular mass and elemental analysis of ES‐414, respectively, exhibited a maximum absorbance at 279 nm, a molecular mass of 414 Da and significantly low carbon content. Against these observations, glycolipids are known to exhibit absorbance at much lower wavelengths, usually in the range of 200–240 nm [85–87], generally possess molecular masses higher than 500 Da [88, 89] and typically contain a high carbon percentage, in the range of 40%–50% [90], making ES‐414 different from common glycolipids. On the contrary, FTIR and thermal analyses revealed properties similar to those of common glycolipids, as ES‐414 displayed the presence of functional groups (hydroxyl and carbonyl) commonly associated with the structure of glycolipids [91, 92], as well as high thermal resistance, remaining stable up to 240°C as observed in TGA [93, 94]. All these observations suggest ES‐414 shares properties with glycolipids yet possesses distinct structural features. These exceptional properties position ES‐414 as a promising candidate for advanced applications across diverse industries and indicate that it requires detailed studies to gain more profound insights into its structural characteristics.

## 5. Conclusion

*E. spinifera* CSF123 produces a biosurfactant molecule that exhibits distinct antibiofilm, antifungal, and antiquorum sensing properties. Detailed characterization of the molecule, named ES‐414, carried out through solubility profiling, UV‐visible spectroscopy, TGA, FTIR, mass spectrometry, elemental composition, TLC, and the Molisch test suggests that it resembles the structure of a glycolipid‐type biosurfactant yet possesses unique properties. This study also unlocks the potential of ES‐414 for transformative applications in bioremediation, pharmaceuticals, and various industrial sectors, leveraging its distinctive properties to deliver innovative solutions.

## Funding

This study was supported by Amrita School of Biotechnology, Amrita Vishwa Vidyapeetham.

## Conflicts of Interest

The authors declare no conflicts of interest.

## Supporting information


**Supporting Information 1** Additional supporting information can be found online in the Supporting Information section. Figure S1: (A) Oil displacement assay: (a) 20 *μ*L of the fungal spent media loaded onto the center of oil exhibiting a zone of clearance of 50 mm, (b) 20 *μ*L of 2% SDS (positive control) showed a displacement zone of 65 mm. (B) Emulsification capacity of the fungal spent media, exhibiting emulsification indices of 33.3% and 66.67% for kerosene and cashew nut shell oil, respectively; 2% SDS was used as a positive control, depicting emulsification indices of 58.6% and 53.3% for kerosene and cashew nut shell oil, respectively. (C) Drop collapse assay demonstrating biosurfactant activity. (a) 20 *μ*L of fungal spent media spread on an oil‐coated well, (b) 20 *μ*L of 2% SDS, used as a positive control, spread on an oil‐coated well. (D) Lipase assay displaying a clear zone around the colonies, indicating enzymatic lipase activity of the fungus *Exophiala spinifera* CSF123. Figure S2: (A) *Exophiala spinifera* CSF123 cultured on a potato dextrose agar plate, exhibiting characteristic blackish‐green pigmentation; (B) Gram′s staining of *E. spinifera* CSF123, revealing the presence of pseudo hyphae; (C) Phylogenetic tree of *E. spinifera* CSF123 with 10 closely related fungi. The tree was made by using the neighbor‐joining method, and evolutionary analyses were conducted using the MEGA11 software tool. Figure S3: Agar well diffusion assay illustrating the antibacterial activity of ES‐414 against *Pseudomonas aeruginosa*, *S. aureus*, and *Chromobacterium violaceum.* (A), (B), and (C), respectively, indicate the positive control, ciprofloxacin (100 *μ*g), negative control (DMSO), and purified ES‐414 (500 *μ*g). Figure S4: Qualitative characterization of the purified biosurfactant showing the presence of carbohydrate and lipid moieties. (A) Molisch test showing a violet ring indicating the presence of carbohydrate moiety in the purified biosurfactant. Sucrose is used as a positive control. (B) Thin‐layer chromatography (TLC) analysis showing the presence of lipid moiety with ES‐414. The sample generated a yellow streak (indicated by the arrow) when exposed to iodine vapors. Cholesterol is used as the positive control. Figure S5: GC‐MS analysis of ES‐414 showing a prominent peak at 124 m/z.

## Data Availability

The data that support the findings of this study are available from the corresponding author upon reasonable request.
