# Supplementary material for: A Glycolipid‐Like Biosurfactant Molecule Derived From an Exophiala spinifera Strain With Significant Antibiofilm, Antifungal, and Antiquorum Sensing Activities
Source: Int J Microbiol. 2026 Jul 23;2026:5630530. doi: 10.1155/ijm/5630530 (PMC13393524; doi:10.1155/ijm/5630530)
Supplement: Supplementary file 1 — Supporting Information 1 Additional supporting information can be found online in the Supporting Information section. Figure S1: (A) Oil displacement assay: (a) 20 μL of the fungal spent media loaded onto the center of oil exhibiting a zone of clearance of 50 mm, (b) 20 μL of 2% SDS (positive control) showed a displacement zone of 65 mm. (B) Emulsification capacity of the fungal spent media, exhibiting emulsification indices of 33.3% and 66.67% for kerosene and cashew nut shell oil, respectively; 2% SDS was used as a positive control, depicting emulsification indices of 58.6% and 53.3% for kerosene and cashew nut shell oil, respectively. (C) Drop collapse assay demonstrating biosurfactant activity. (a) 20 μL of fungal spent media spread on an oil‐coated well, (b) 20 μL of 2% SDS, used as a positive control, spread on an oil‐coated well. (D) Lipase assay displaying a clear zone around the colonies, indicating enzymatic lipase activity of the fungus Exophiala spinifera CSF123. Figure S2: (A) Exophiala spinifera CSF123 cultured on a potato dextrose agar plate, exhibiting characteristic blackish‐green pigmentation; (B) Gram′s staining of E. spinifera CSF123, revealing the presence of pseudo hyphae; (C) Phylogenetic tree of E. spinifera CSF123 with 10 closely related fungi. The tree was made by using the neighbor‐joining method, and evolutionary analyses were conducted using the MEGA11 software tool. Figure S3: Agar well diffusion assay illustrating the antibacterial activity of ES‐414 against Pseudomonas aeruginosa, S. aureus, and Chromobacterium violaceum. (A), (B), and (C), respectively, indicate the positive control, ciprofloxacin (100 μg), negative control (DMSO), and purified ES‐414 (500 μg). Figure S4: Qualitative characterization of the purified biosurfactant showing the presence of carbohydrate and lipid moieties. (A) Molisch test showing a violet ring indicating the presence of carbohydrate moiety in the purified biosurfactant. Sucrose is used as a positive con [file IJM-2026-5630530-s001.docx]

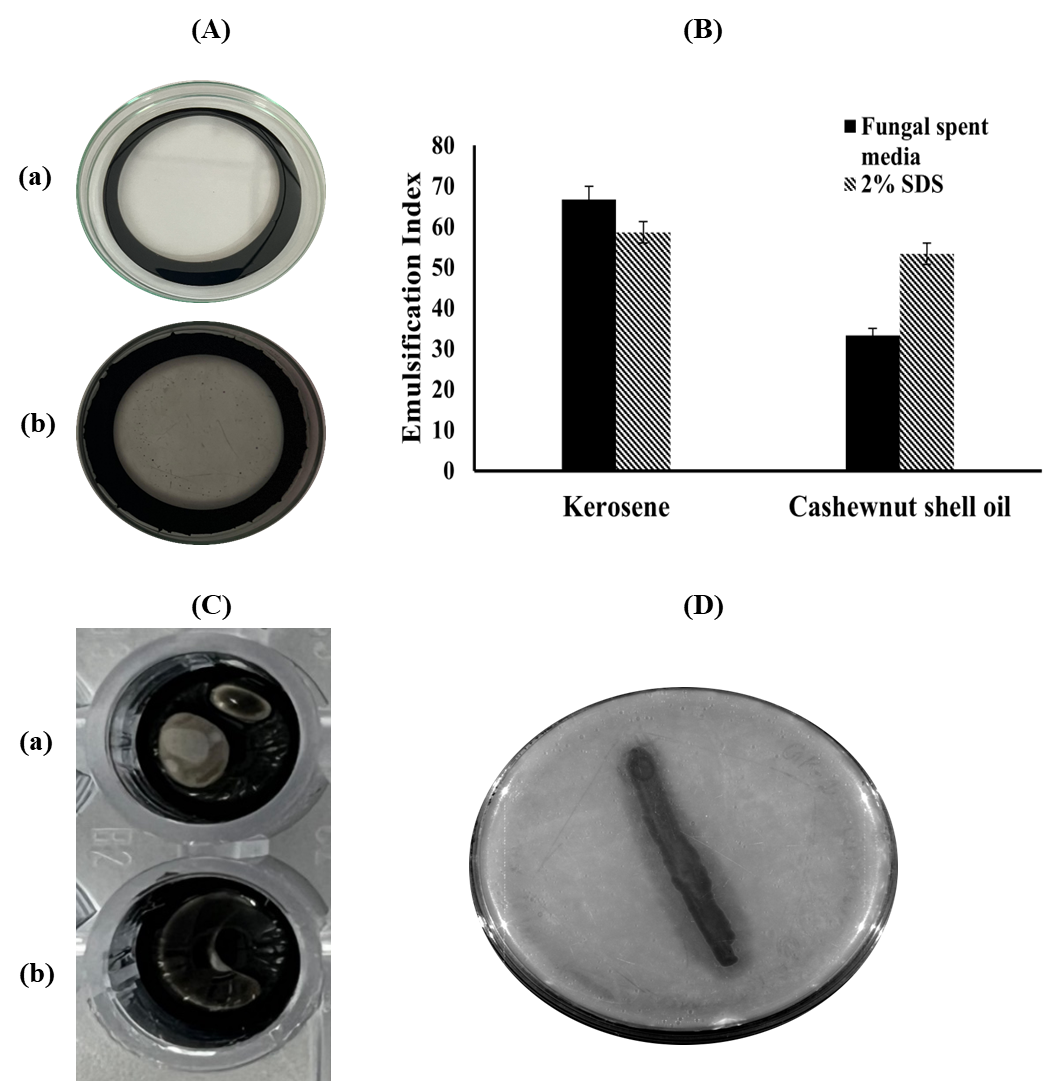


Figure S1: (A) Oil Displacement Assay: (a) 20 µL of the fungal spent media loaded onto the center of oil exhibiting a zone of clearance of 50 mm, (b) 20 µL of 2% SDS (positive control) showed a displacement zone of 65 mm. (B) Emulsification capacity of the fungal spent media, exhibiting emulsification indices of 33.3 and 66.67% for kerosene and cashew nut shell oil, respectively; 2% SDS was used as a positive control, depicting emulsification indices of 58.6 and 53.3% for kerosene and cashew nut shell oil, respectively. (C) Drop collapse assay demonstrating biosurfactant activity. (a) 20 µL of fungal spent media spread on an oil-coated well, (b) 20 µL of 2% SDS, used as a positive control, spread on an oil-coated well. (D) Lipase assay displaying a clear zone around the colonies, indicating enzymatic lipase activity of the fungus *Exophiala spinifera* CSF123.


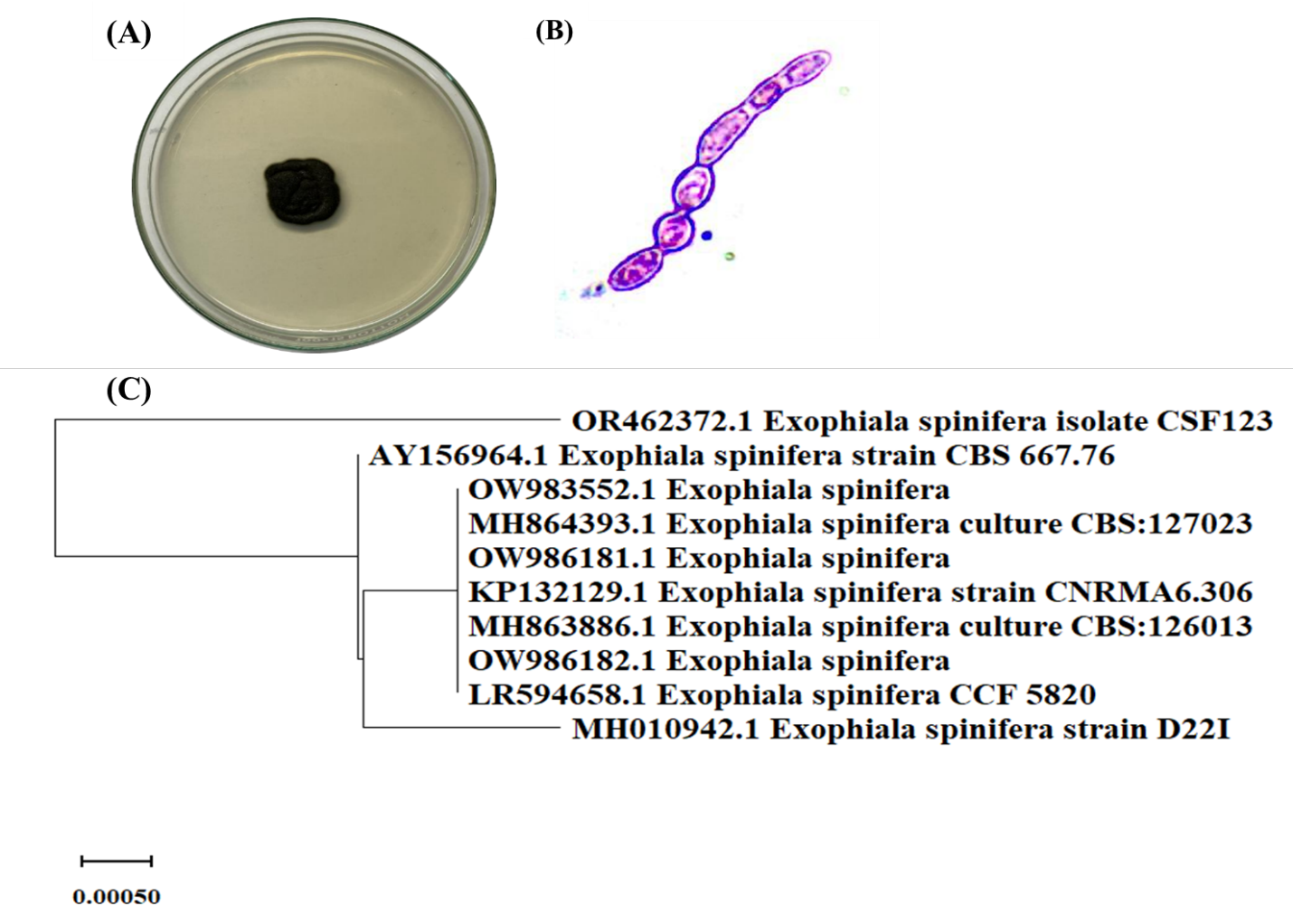


Figure S2: (A) *Exophiala spinifera* CSF123 cultured on a potato dextrose agar plate, exhibiting characteristic blackish-green pigmentation; (B) Gram’s staining of *Exophiala spinifera* CSF123, revealing the presence of pseudo hyphae; (C) Phylogenetic tree of *Exophiala spinifera* CSF123 with ten closely related fungi. The tree was made by using the neighbour-joining method, and evolutionary analyses were conducted using the MEGA11 software tool.


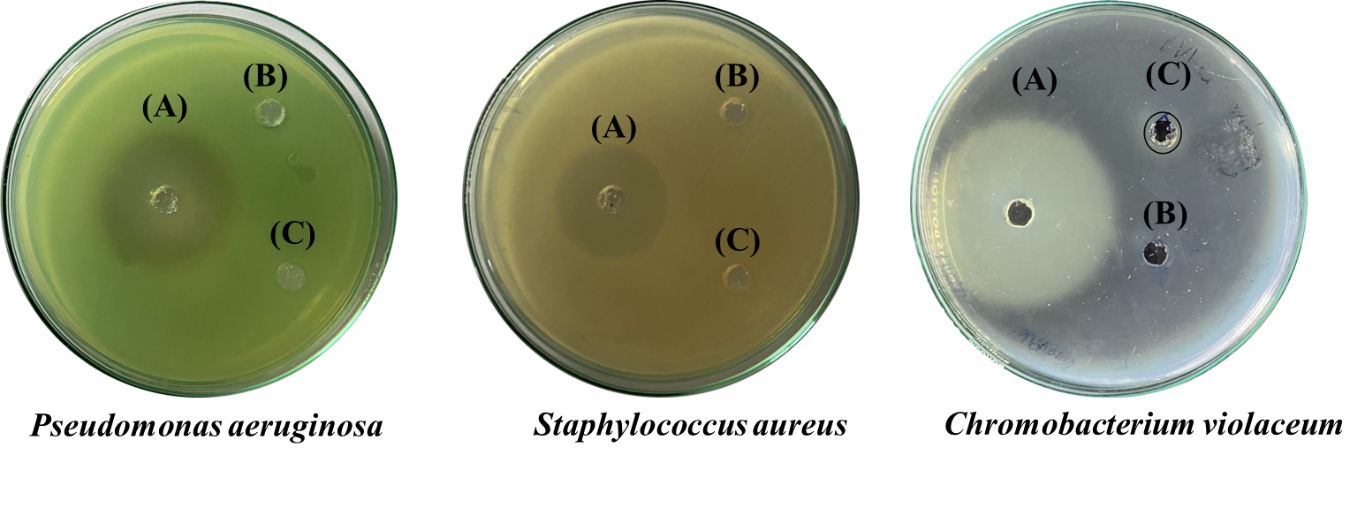


Figure S3: Agar well diffusion assay illustrating the antibacterial activity of ES-414 against *Pseudomonas* *aeruginosa*, *Staphylococcus* *aureus* and *Chromobacterium violaceum.* (A), (B) and (C) respectively indicate the positive control [ciprofloxacin (100 μg)], negative control (DMSO) and purified ES-414 (500 μg).


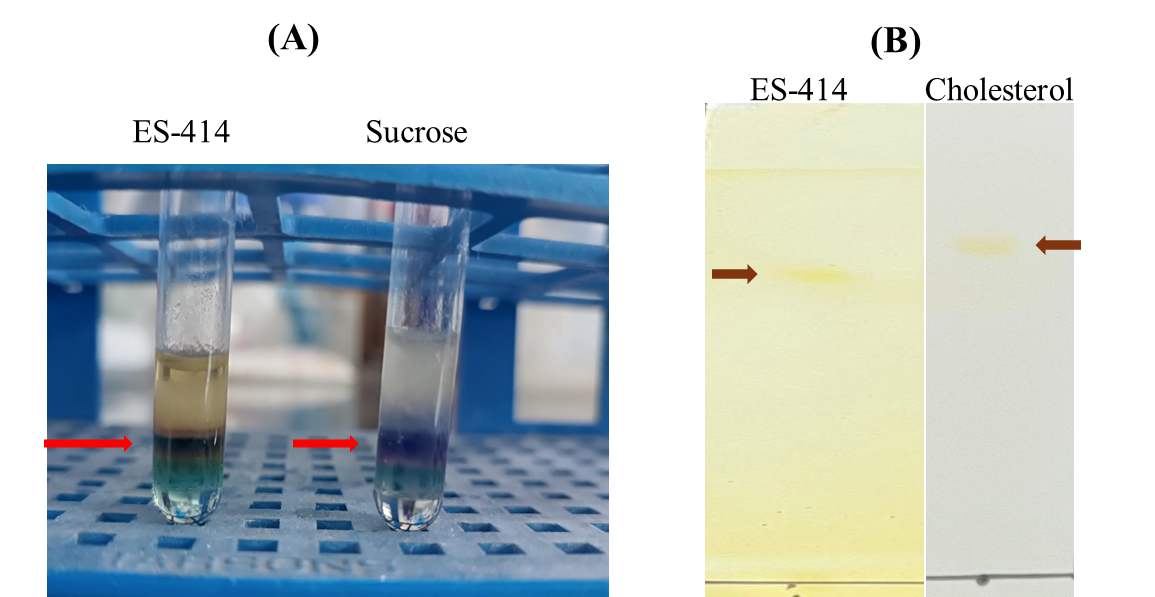


Figure S4: Qualitative characterization of the purified biosurfactant showing the presence of carbohydrate and lipid moieties. (A) Molisch test showing a violet ring indicating the presence of carbohydrate moiety in the purified biosurfactant. Sucrose is used as a positive control. (B) Thin-layer chromatography (TLC) analysis showing the presence of lipid moiety with ES-414. The sample generated a yellow streak (indicated by the arrow) when exposed to iodine vapors. Cholesterol is used as a positive control.

**190**

**70**

**90**

**110**

**4**

**x10**

**0**

**2**

**4**

**6**

**124.040**

**208.140**

**137.050**

**Mass-to-Charge (m/z)**

**230**

**Intensity**

**210**

**50**

**130**

**150**

**170**

Figure S5: GC-MS analysis of ES-414 showing a prominent peak at 124 m/z.
